# Metabolic engineering of *Nicotiana benthamiana* for production of curcuminoids

**DOI:** 10.1007/s00425-026-05014-x

**Published:** 2026-05-11

**Authors:** Rafael González-Castro, Enrique Ramírez-Chávez, Jorge Molina-Torres, Brisia Alejandra Aguilar-Barragán, Miguel Angel Gómez-Lim

**Affiliations:** Centro de Investigación y Estudios Avanzados del Instituto Politécnico Nacional, Unidad Irapuato, 36824 Guanajuato México Irapuato,

**Keywords:** Agroinfiltration, Plant biotechnology, Heterologous expression, Polyketide synthases, Metabolic engineering, Secondary metabolites

## Abstract

**Main conclusion:**

*Nicotiana benthamiana* plants transiently transformed with the C4 vector are capable to produce curcumin, demethoxycurcumin and bisdemethoxycurcumin in reasonable yields.

**Abstract:**

Curcuminoids are valuable bioactive compounds whose heterologous production remains limited by precursor availability, cytotoxic intermediates and imbalanced metabolite ratios in microbial systems. In this study, we employed *Nicotiana benthamiana* as a platform for the transient expression of turmeric polyketide synthases, including diketide–CoA synthase (DCS) and curcumin synthases 1–3 (CURS1–3). Using *Agrobacterium tumefaciens* and a polycistronic expression vector (C4), we achieved the coordinated production of curcumin [187.7 ± 4.6 µg/g dry weight (DW)], demethoxycurcumin (41.8 ± 2.3 µg/g DW) and bisdemethoxycurcumin (31 ± 4.6 µg/g DW). Notably, the relative proportions of these metabolites closely matched those found in turmeric rhizomes. Metabolite identity was confirmed by chromatographic and spectroscopic analyses. These results demonstrate that plant-based transient expression systems can overcome key limitations of microbial platforms and enable the production of curcuminoids in physiologically relevant ratios.

**Supplementary Information:**

The online version contains supplementary material available at 10.1007/s00425-026-05014-x.

## Introduction

Curcuminoids are diarylheptanoids (C6–C7–C6) compounds naturally produced in the rhizome of turmeric plant (*Curcuma longa*) (Jayaprakasha et al. 2005). The rhizome contains a mixture of curcuminoids in which curcumin is the main active chemical constituent, although other curcuminoids such as demethoxycurcumin and bisdemethoxycurcumin are also majoritarian components of this mixture (Srinivasan 1953). Curcuminoids, naturally constitute approximately 5% of the dry weight of dried rhizome in a mixture percentage of curcumin (77%), demethoxycurcumin (18%) and bisdemethoxycurcumin (5%), respectively (Bagchi 2012). These metabolites have been reported to possess diverse therapeutic properties, for example: anti-bacterial (Negi et al. 1999), anti-carcinogenic (Akter et al. 2025), anti-cholesterolemic (Kim and Kim 2010), anti-inflammatory (Huang et al. 1991), anti-oxidant (Menon and Sudheer 2007) or anti-diabetic effects (Kim et al. 2009).

Curcumin, demethoxycurcumin, and bisdemethoxycurcumin have been extensively studied, and although these metabolites exhibit potent therapeutic effects individually, various studies have shown that when administered together they exhibit a stronger synergistic effect (Wei et al. 2021).

**Fig. 1:**
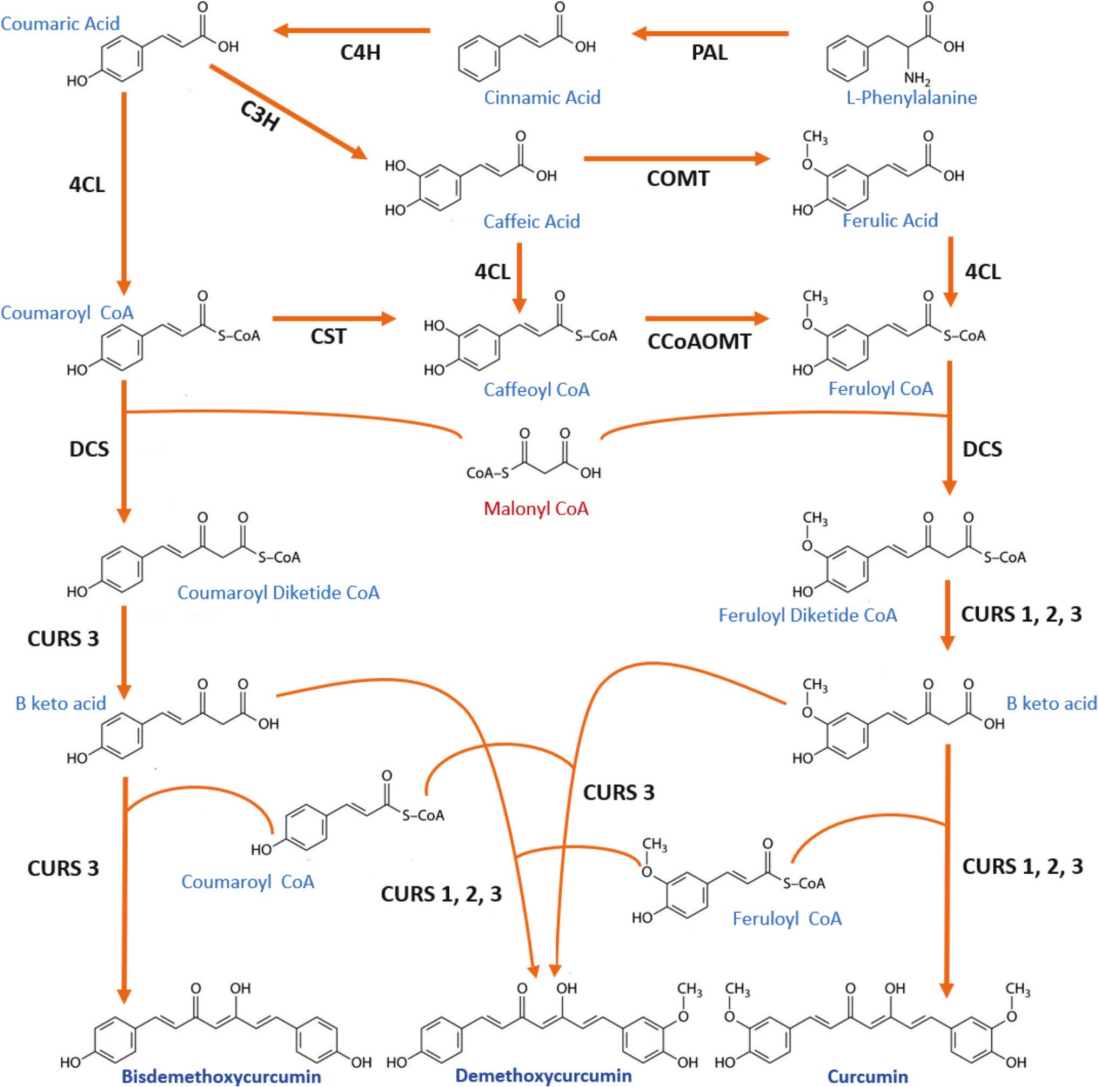
Metabolic pathway of curcuminoid biosynthesis in *C. longa* initiates with the conversion of phenylalanine into cinnamic acid via the action of phenylalanine ammonia-lyase. Cinnamic acid is subsequently hydroxylated by cinnamate-4-hydroxylase (C4H) to yield coumaric acid. This compound is then activated to coumaroyl-CoA through the enzymatic activity of 4-coumarate-CoA ligase (4CL). A series of enzymatic steps involving p-coumaroyl shikimate transferase, p-coumaroyl 5-O-shikimate 3′-hydroxylase (C3H), and caffeoyl-CoA O-methyltransferase (CCoAOMT) further transform coumaroyl-CoA into feruloyl-CoA. DCS subsequently condenses coumaroyl-CoA and feruloyl-CoA with malonyl-CoA to form diketide-CoAs. In the final step, CURSs facilitate the condensation of these diketide-CoAs with either coumaroyl-CoA or feruloyl-CoA, resulting in the synthesis of distinct curcuminoids such as bisdemethoxycurcumin, demethoxycurcumin, and curcumin. Image was created using ACD/ChemSketch program for molecular structures

In turmeric, these compounds are produced by polyketide synthases in the phenylpropanoid pathway. The chemical structure of these metabolites consists of two phenylpropanoid units derived from phenylalanine and connected by a central carbon derived from malonyl-coenzyme A (malonyl-CoA). Phenylalanine ammonia lyase, p-coumaroyl shikimate transferase, p-coumaroyl quinate transferase, caffeic acid O-methyltransferase, and caffeoyl-CoA O-methyltransferase are enzymes directly involved in the first part of this metabolic route. Nevertheless, another four polyketide synthases enzymes are involved in curcuminoid production, in the second part of the metabolic route: DCS, CURS 1, CURS 2 and CURS 3 (Katsuyama et al. 2009a, b). DCS, catalyzes the formation of coumaroyl-diketide-CoA and feruloyl-diketide-CoA from coumaroyl-CoA and feruloyl-CoA, respectively. In this part of the route, malonyl-CoA is used as an extensor molecule to generate the products. Subsequently, the CURS enzymes catalyze two reactions. First, they catalyze the hydrolysis of diketide-CoA into β-keto acids. Second, by using the corresponding β-keto acid and other molecule of coumaroyl-CoA or feruloyl-CoA as substrates, they catalyze the formation of curcumin, demethoxycurcumin or bisdemethoxycurcumin (Fig. 1).

The natural asymmetric production of curcumin, demethoxycurcumin, and bisdemethoxycurcumin (77:18:5, respectively) is probably attributable to the differential substrate affinity exhibited by the CURS enzymes. For example, different analyses revealed that CURS1 and CURS2 preferred feruloyl-CoA as the substrate, while CURS3 used both, feruloyl-CoA and p-coumaroyl-CoA (Katsuyama et al. 2009a, b).

Curcuminoids, like many other secondary metabolites in plants, accumulate in tissues at very low concentrations over an extended period of time and are difficult and expensive to isolate. On the other hand, their structural complexity makes them difficult to synthesize chemically. This combination of factors, plus their demonstrated therapeutical effects, have led to an increased interest over the last 15 years to develop biosynthesis in heterologous systems. Ideally the system should grow on inexpensive substrates, be of easy manipulation, be susceptible for large scale production and have a simple purification process. Micro-organisms have been employed for biosynthesis (Lussier et al. 2012) but a considerable number of challenges have emerged over time. For example, the primary challenge lies in the adverse impact on microbial fitness and metabolic profile, since the majority of the enzymes required for curcuminoid biosynthesis are not present in the model host (Katsuyama et al. 2008). In addition, the inherently very low bioavailability of malonyl-CoA in bacterial and yeast systems, as well as the significant toxicity of biosynthetic intermediates—such as ferulic acid—to the production host are problematic to resolve. Finally, the curcuminoids are produced in a ratio that does not reflect the natural proportion. Nevertheless, in recent years, some efforts to overcome these problems had led to the development of different strategies. For example, Chen et al. (2024) used engineered *Escherichia coli* achieving yields of 696 mg/L of curcumin. Therefore, Incha et al. (2020) produced demethoxycurcumin utilizing *Pseudomonas putida* for the inner ability of this organism to utilize both ferulic and p-coumaric acids as substrates. Curcuminoid production has also been achieved in fungus and yeasts. Kan et al. (2019) developed a recombinant *Aspergillus oryzae* strain expressing the curcuminoid producer enzyme Curcuminoid synthase (CUS), achieving a curcumin production of 64 μg/plate. Palmer et al. (2020) inserted and overexpressed several genes from the phenylpropanoid route in *Yarrowia lipolytica* (*Y. lipolytica*), including the curcuminoid synthase gene from *Oryza sativa* (*O. sativa*) and obtained naringenin, resveratrol and bisdemethoxycurcumin with a yield of 0.17 mg/L for the latter. Rainha et al. (2024) constructed a *Saccharomyces cerevisiae* strain capable of synthesizing curcumin directly from glucose integrating 4-hydroxyphenylacetate 3-monooxygenase, caffeic acid O-methyltransferase, feruloyl-CoA synthetase, DCS and CURS1 into the metabolome of the organism. The engineered strain obtained a production yield of 4.2 ± 0.6 mg/L.

With regard to curcuminoid production in plants, there are very few precedents, for example, the work elaborated by Singh et al. (2021). They successfully produced curcumin and its glucoside in *Atropa belladonna* (*A. belladonna*) hairy root culture. They expressed DCS and CURS3 from *C. longa*, along with the glucosyltransferase CaUGT2. The curcumin yield was 180.62 ± 4.7 μg/g DW and 32.63 ± 2.27 μg/g DW of curcumin monoglucoside. Martí-Botella (2022) produced curcuminoids in *Nicotiana benthamiana* (*N. benthamiana)* using a multigene strategy in which they co-expressed DCS1 and CURS3 enzymes using a chimeric construct derived from tobacco etch virus, resulting in a curcumin accumulation of 22 ± 4 μg/g in *N. benthamiana* leaf DW after at 11 day post-inoculation.

Many different strategies have been used for heterologous curcuminoid production, nevertheless, no reported platform so far has yielded the ratio of the three main curcuminoids found in nature (77:18:5). It is not clear whether this ratio is important to realize all the therapeutic effects of curcuminoids but considering that they have a strong synergistic effect when administered together (Wei et al. 2021), it is likely that the right proportion of curcuminoids it is important to keep to attain full benefits. Even though very few published reports (Singh et al. 2021), we believe that plants represent an attractive alternative for the production of curcuminoids. Since the genes of the phenylpropanoid pathway are already present in plants (Jeandet et al. 2012), it may be necessary to introduce only few relevant genes. In addition, plants represent a significantly lower cross-pathogen risk than animal or microbial cells. For these reasons, in this study, we used plants as platform for the heterologous production of curcuminoids using transient expression. *N. benthamiana* plants were transformed with four exogenous genes (DCS, CURS1, CURS2 and CURS3) introduced in a polycistronic arrangement. When more than one gene is required to obtain a specific product, many researchers prefer to introduce each gene as a separate cassette. In our case, we decided to employ a polycistron-like construct containing all four genes separated by ubiquitin linkers which are removed by the cell machinery and the fused proteins moieties are released in free forms. Furthermore, synthesis of a protein as an ubiquitin fusion can significantly enhance its accumulation (Hondred et al. 1999). The results obtained demonstrated that the transiently transformed plants were capable of producing the desired curcuminoids at the natural proportion.

## Materials and methods

### Vector design and *Agrobacterium tumefaciens* transient transformation

**Fig. 2:**
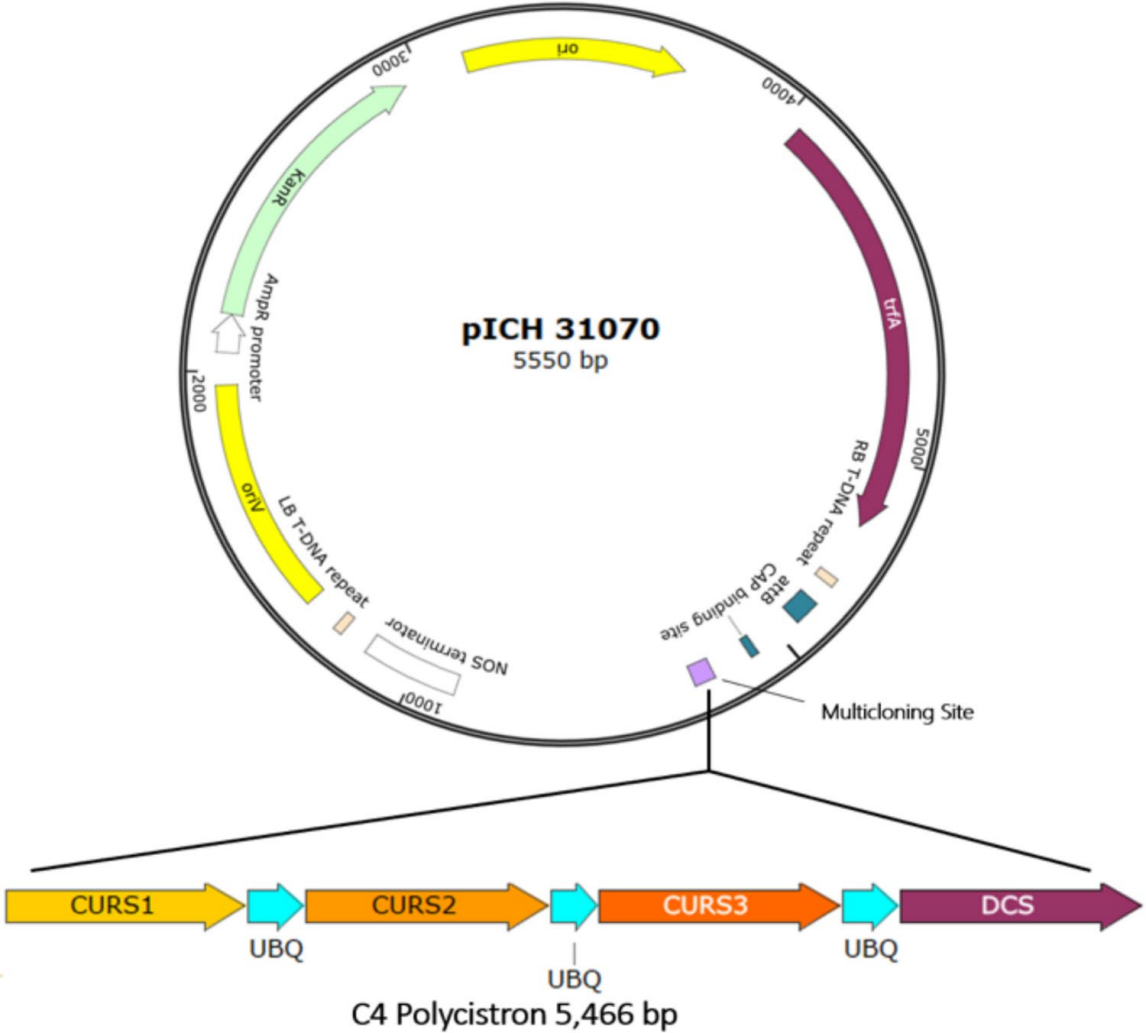
The C4 polycistron was inserted into the multiple cloning site of the pICH31070 vector. This polycistronic construct contains the coding sequences of the four enzymes required for curcuminoid biosynthesis: DCS, CURS1, CURS2, and CURS3. The coding regions of these enzymes are separated by ubiquitin-encoding sequences. The C4 vector was used in combination with the pICH10881 and pICH4851 vectors for the heterologous production of curcuminoids using the Magnifection protocol (as described in the Methodology section)

The DNA coding sequence (GenBank: AB495006.1, UniProt: C0SVZ6 · CURS1_CURLO, UniProt: C6L7V8 · CURS2_CURLO and UniProt: C6L7V9 · CURS3_CURLO) for each of the curcuminoid-producing enzymes was inserted into the cloning vector pICH31070, each flanked by coding sequences of ubiquitin (UniProt: P0CG47 · UBB_HUMAN) (Fig. 2). The entire coding region was flanked by BsaI restriction sites at both the 5′ and 3′ ends. The sequences were synthesized by GenScript™ (New Jersey, USA) and designated as C4. C4 vector was inserted into electrocompetent *Agrobacterium tumefaciens* GV3101, transformed bacteria were recovered and plated onto YEB agar medium supplemented with rifampicin 50 mg/mL and kanamycin at 50 mg/mL. The plates were incubated at 28 °C for 48 h for bacterial growth.

### *N. benthamiana* agroinfiltration and gene expression

For transient expression by the magnifection system (Marillonnet et al. 2004), *A. tumefaciens* GV3101 strain, carrying vectors C4-pICH31070 (hereafter referred to as C4), pICH10881, pICH4851, or pICH6891 (all vectors, except C4, were generously provided by Dr. Yuri Gleba, NOMAD Bioscience GmbHTM, Munich, Germany) were grown into 5 mL YEB medium supplemented with kanamycin (50 µg/mL) and incubated at 28 °C for 48 h with shaking (200 rpm). 2 mL from each primary culture were transferred into separate 1 L Erlenmeyer flasks containing 230 mL YEB medium with kanamycin (50 µg/mL) and carbenicillin (100 µg/mL) and incubated at 28 °C for 24 h with shaking until an OD₆₀₀ of 1.5 is reached. According to Marillonnet et al. (2004), *A. tumefaciens* GV3101 strains carrying individually C4, pICH10881, pICH4851, or pICH6891 vectors were incubated in YEB medium at 28 °C. Each culture was centrifugated (3000 × *g*) and the pellet of each culture was resuspended in 250 mL of agroinfiltration buffer (10 mM MES pH 5.5, 10 mM MgSO_4_). For the successful synthesis of the product of interest, the vectors must be co-cultivated at the same molar concentration, as reported by Marillonnet et al. (2004). Cultures were mixed (C4–pICH10881–pICH4851 or pICH6891–pICH10881–pICH4851) and the bacterial suspension was supplemented with 200 μM of acetosyringone and incubated for 2 h at room temperature. *N. benthamiana* plants were grown in a controlled-ambient chamber (25 °C and 16/8 light/darkness) and 6-week-old plants were infiltrated with bacterial suspension using a syringe without needle. Sixty plants per batch were infiltrated and incubated under controlled conditions (25 °C and 16/8 light/darkness). Plants infiltrated for GFP production and plants infiltrated with the C4 vector for curcuminoid production were kept in a separate incubator to avoid a possible cross-contamination. Monitoring of plants started 4–5 day post-agroinfiltration. Examination was performed under UV light to assess transgene expression. Due to the accumulation of curcuminoids on the leaves of the transiently transformed plants, they were harvested 15–21 day post-agroinfiltration. The agroinfiltrated leaves from each plant were collected, frozen in liquid nitrogen, macerated, and freeze-dried for further analysis.

### Extraction of components from transiently transformed *N. benthamiana* leaves extract using a Soxhlet system

For curcuminoid extraction, 15 g of dried leaves from transformed *N. benthamiana* plants were used employing a Soxhlet system (Büchi™ E-18) with the addition of 150 mL of ultrapure-grade ethanol. Extraction columns were covered with aluminum foil to prevent light-induced degradation of potential curcuminoids in the extract and was heated to 80 °C and subsequently condensed. The 30 min boiling-condensation cycle was repeated five times to maximize the extraction of compounds present in plant tissue. 150 mL of ethanol extract were recovered for subsequent separation.

### Separation of components in transiently transformed *N. benthamiana* plants extract using a hexane–ethyl acetate eluotropic series

For the separation process, 50 mL of the sample obtained by Soxhlet extraction were loaded onto a 50 × 3 cm glass column packed with silica gel (60–120 mesh) as the stationary phase. 50 mL of hexane were passed through the column to elute the less polar components of the sample. A hexane–ethyl acetate eluotropic gradient was then employed for mobile phase. Eluotropic series ranging from 100% hexane to 100% ethyl acetate were used, and the eluates corresponding to each hexane–ethyl acetate ratio were collected.

### High performance thin layer chromatography to determine the separation pattern of curcuminoids

Pattern separation for curcuminoids was determined using a preparative high performance thin layer chromatography Silica gel 60 F₂₅₄ plates (2 mm thickness, 20 × 20 cm; Merck™ 105717). A commercial curcuminoid mixture at a concentration of 4 mg/mL was used as a positive control and was sprayed across the width of the plate at a height of 2 cm from the base. The total volume of the applied sample was 5 mL. The mobile phase consisted of a chloroform/methanol mixture (45:5 v/v). The plate was then dried in total darkness. Separation of the components was subsequently visualized under UV light at 365 nm. The silica corresponding to each of the three curcuminoid bands was carefully scraped from the plate and collected separately in 2 mL Eppendorf tubes for curcuminoid extraction from the silica. To recover the embedded curcuminoids, 1 mL of absolute ethanol was added to each silica sample. The entire process was carried out in the dark to prevent degradation of the curcuminoids in solution. To enhance extraction efficiency, the samples were vortexed for 2 min and then subjected to a 10 min ultrasonic bath. Once the separation pattern for the positive control was established, the same methodology was applied to separate the curcuminoid components from samples derived from the hexane–ethyl acetate eluotropic series. For sample application, 10 µL of each sample were sprayed onto high performance thin layer chromatography plates using a CAMAG™ ATS-4 automated sampler.

### Curcuminoid detection by reverse-phase high performance liquid chromatography

Curcuminoids present in a commercial mixture were employed preliminary as a positive control, the detection was carried out using reverse-phase high-performance liquid chromatography (RP-HPLC). A 50 μL aliquot of the commercial curcuminoid mixture, dissolved in ultrapure-grade methanol at a concentration of 1 mg/mL, was injected into an Agilent™ C18 column (4.6 × 250 mm). The mobile phase consisted of acetonitrile with 10 mM Na₂HPO₄–H₃PO₄ buffer (pH 5.0). The flow rate was 1 mL per min. Detection parameters were adjusted to a potential of 0.9 V and a wavelength of 370 nm. The analysis was carried out over a 20 min run time. Once the detection parameters for the positive control were established, the same methodology was applied to analyze curcuminoids produced in the transiently transformed plants and previously isolated and separated via preparative TLC.

### Curcuminoid detection by electrospray mass spectrometry in extracts from transiently transformed *N. benthamiana* plants

Curcuminoid candidates extracted from silica of preparative TLC were analyzed by mass spectrometry in a LCQ Fleet™ ion trap mass spectrometer equipment. The analysis employed mass spectrometry electrospray ionization in negative ion mode [(ESI–MS (−)]. Both experimental and control samples were loaded separately into an Agilent C18 column (100 × 2.1 mm). An isocratic elution system was employed, consisting of two mobile phases: A (acetonitrile with 0.1% formic acid) and B (water with 0.1% formic acid). Each sample was analyzed over a 15 min run until reaching a solvent composition of 43% A/57% B.

### Curcuminoid quantification by RP-HPLC

Quantification of heterologously produced curcuminoids was achieved by analyzing samples derived from three independent transient transformation experiments conducted separately. Mean and standard deviation (±) from those samples were obtained using JMP Statistical Discovery program. The quantification experiment was carried out on a Shimadzu HPLC system equipped with a Zorbax Eclipse XOB-C18 column (4.6 × 150 mm, 3.5 µm particle size; Merck, Germany) and an SPD-20A photodiode array detector (Shimadzu Scientific Instruments, Columbia, MD, USA). The mobile phase consisted of water and acetonitrile (55:45, v/v) with 0.1% acetic acid. Separation was performed at a flow rate of 1.0 mL/min, and compounds were detected at 425 nm.

## Results

### Curcuminoid heterologous production by transient expression in *N. benthamiana* plants

**Fig. 3:**
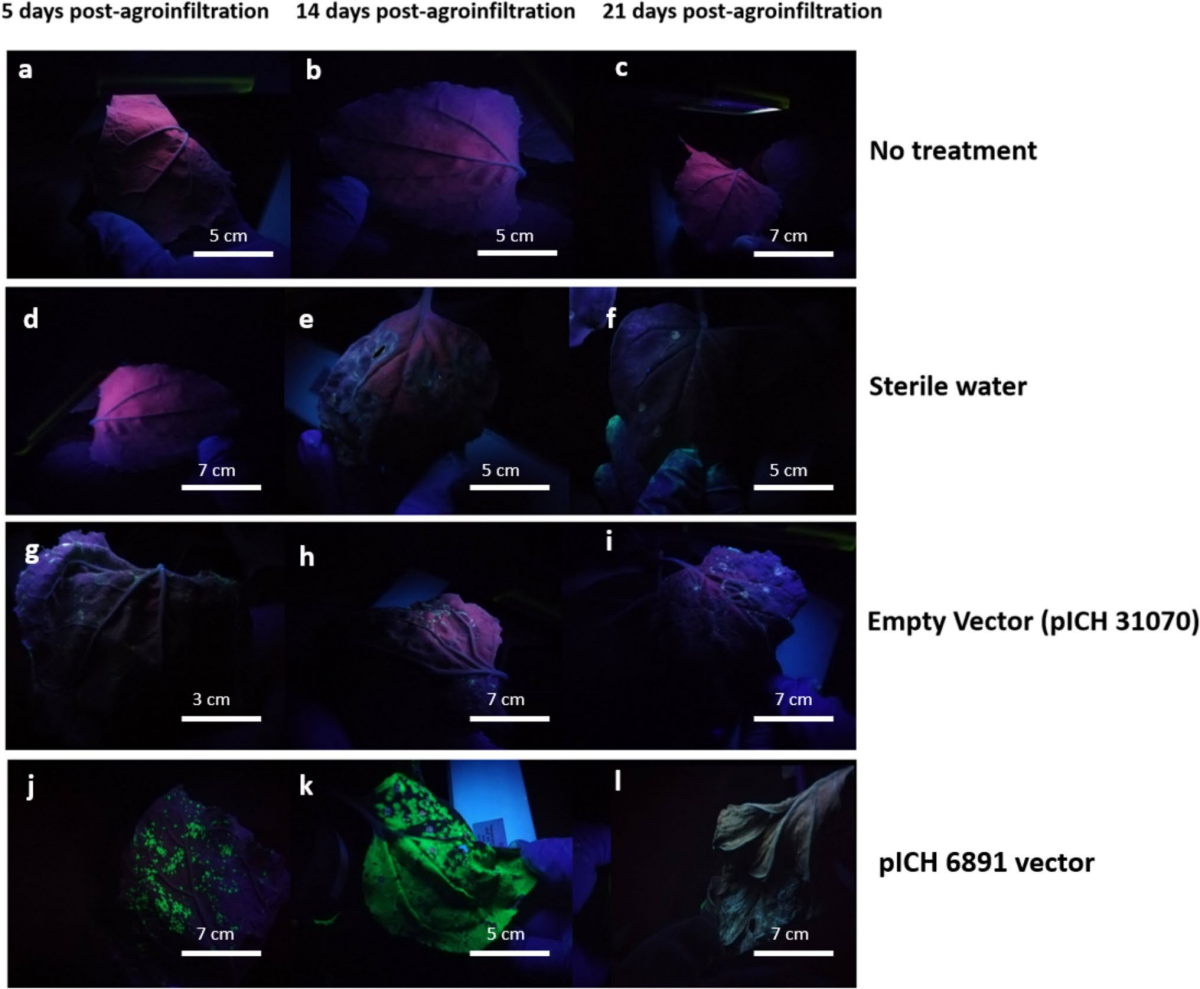
Leaves of *N. benthamiana* control plants used to assess the transient expression of curcuminoids under different treatments were photographed at 5, 14 and 21 days post-infiltration. Plants that did not receive any treatment (**a**–**c**), as well as those infiltrated with water (**d**–**f**) or with the empty vector (**g**–**i**), did not produce curcuminoids. In contrast, the positive expression control plants produced GFP as early as 5 days post-agroinfiltration (**j**–**l**). Plants were observed under UV light at 365 nm

**Fig. 4:**
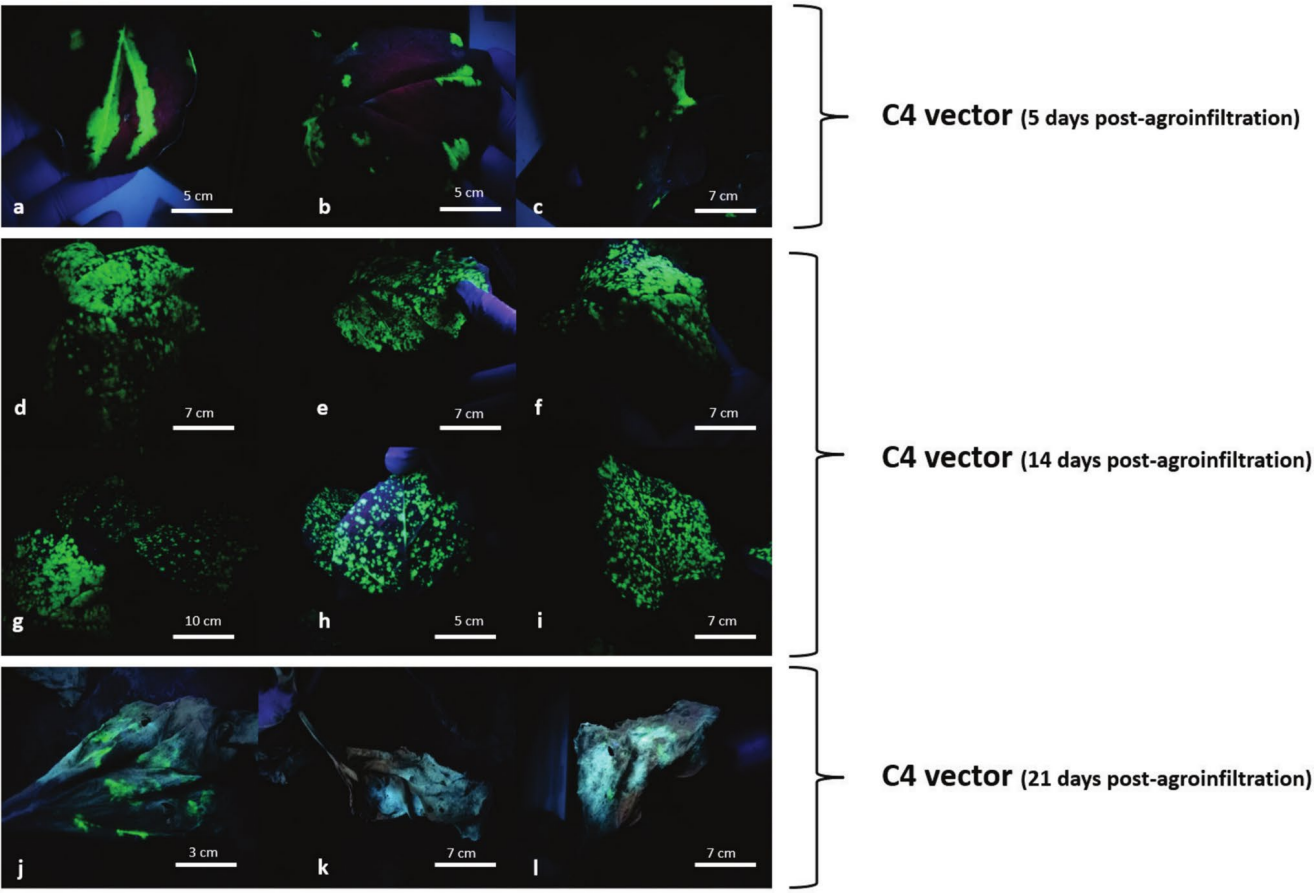
Leaves of *N. benthamiana* plants agroinfiltrated with the C4 vector were photographed at different days post-agroinfiltration. The plants exhibit luminescence very similar to that observed in GFP-expressing plants when viewed under ultraviolet light at 365 nm. Luminescence begins to appear between 4–5 days post-agroinfiltration in a band-like pattern (**a**–**c**), and subsequently spreads across the entire leaf (**d**–**i**). By the third week post-agroinfiltration, the green luminescence begins to shift to a bluish hue (**j**–**l**)

Since curcuminoid biosynthesis requires only four specific enzymes, their heterologous production in *N. benthamiana* plants should theoretically be sufficient by introducing only these enzymes into the plant genome, DCS, CURS1, CURS2, and CURS3 in the pICH31070 vector (Fig. 2). For transient transformation positive control, GFP protein was employed. Negative controls included non-infiltrated *N. benthamiana* plants, plants agroinfiltrated with vectors with no insert and plants infiltrated with water. Plants used as negative controls did not produce any detectable products (Fig. 3a–i), whereas the GFP-expressing plants displayed characteristic bioluminescence observed under 365 nm UV light on the 5th day post-agroinfiltration (Fig. 3j–l). Transiently transformed leaves began to senesce around 17 day post-agroinfiltration likely due to GFP overaccumulation (Fig. 3l). Curcuminoids exhibit fluorescence when exposed to UV light and the *N. benthamiana* plants transformed with the C4 vector exhibited fluorescence under UV light, similar in appearance to that observed in the GFP-expressing plants (Fig. 4a–c). The peak fluorescence in C4-transiently transformed plants was reached at 14 day post-agroinfiltration (Fig. 4d–i). Nevertheless, if the plants were not harvested by day 19 post-agroinfiltration, the fluorescence began to shift from a bright green to a bluish-white coloration and leaves began to senescence (Fig. 4j–l).

### Separation of the components extracted from *N. benthamiana* transiently transformed plants

**Fig. 5:**
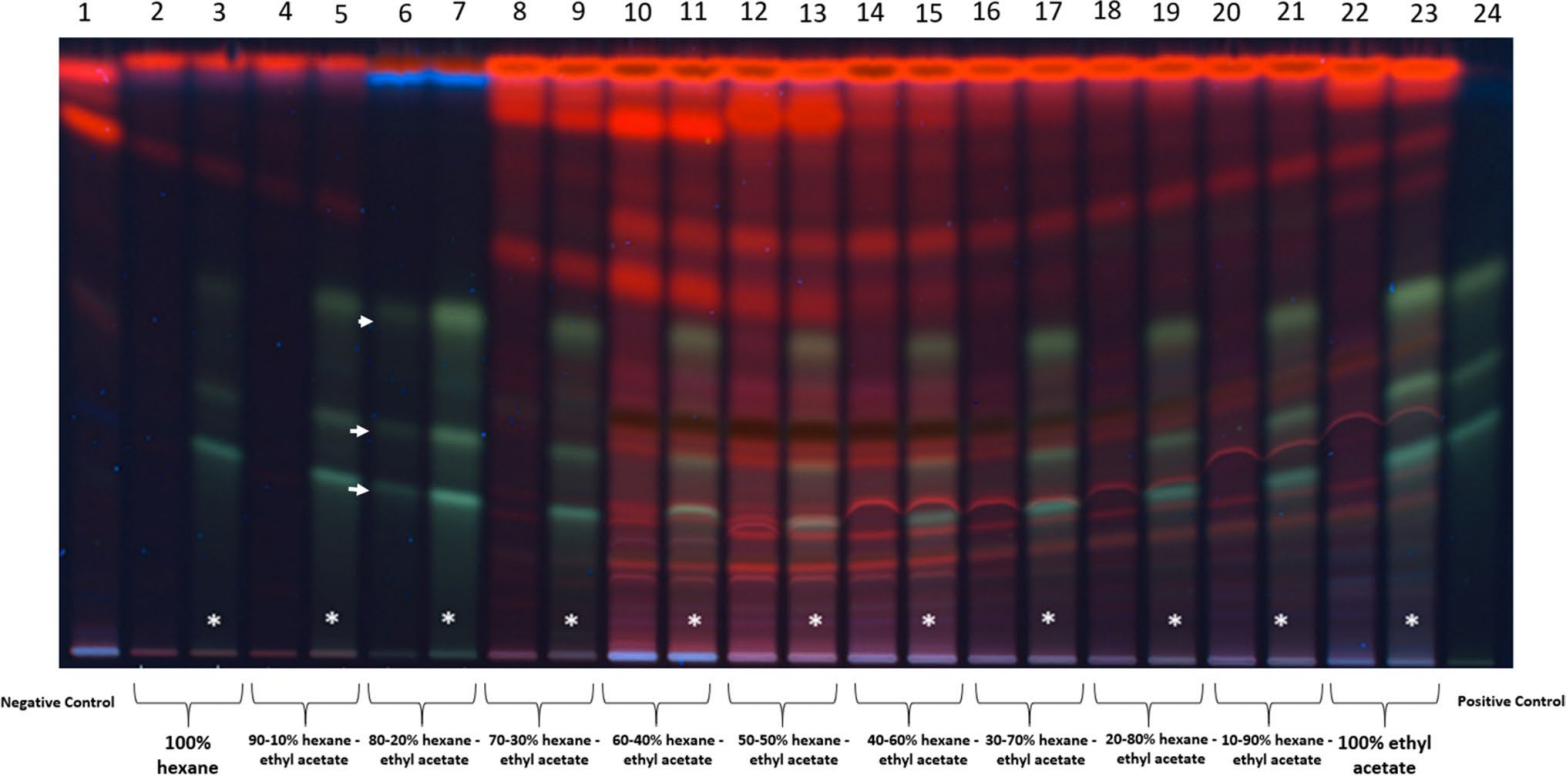
Eluotropic series of hexane–ethyl acetate were used for the separation of components from the *N. benthamiana* extract. The samples obtained after the hexane–ethyl acetate eluotropic series were supplemented with the commercial curcuminoid mixture (white asterisks) to compare banding patterns and detect possible curcuminoid presence. Extracts of wild type *N. benthamiana* was used as negative control and a curcuminoid mixture was utilized as a positive control. Fraction 80–20% hexane–ethyl acetate exhibited a banding pattern similar to that observed in the sample supplemented with the curcuminoid mixture and in the positive control (white arrows). TLC plaque was observed under UV light at 365 nm

**Fig. 6:**
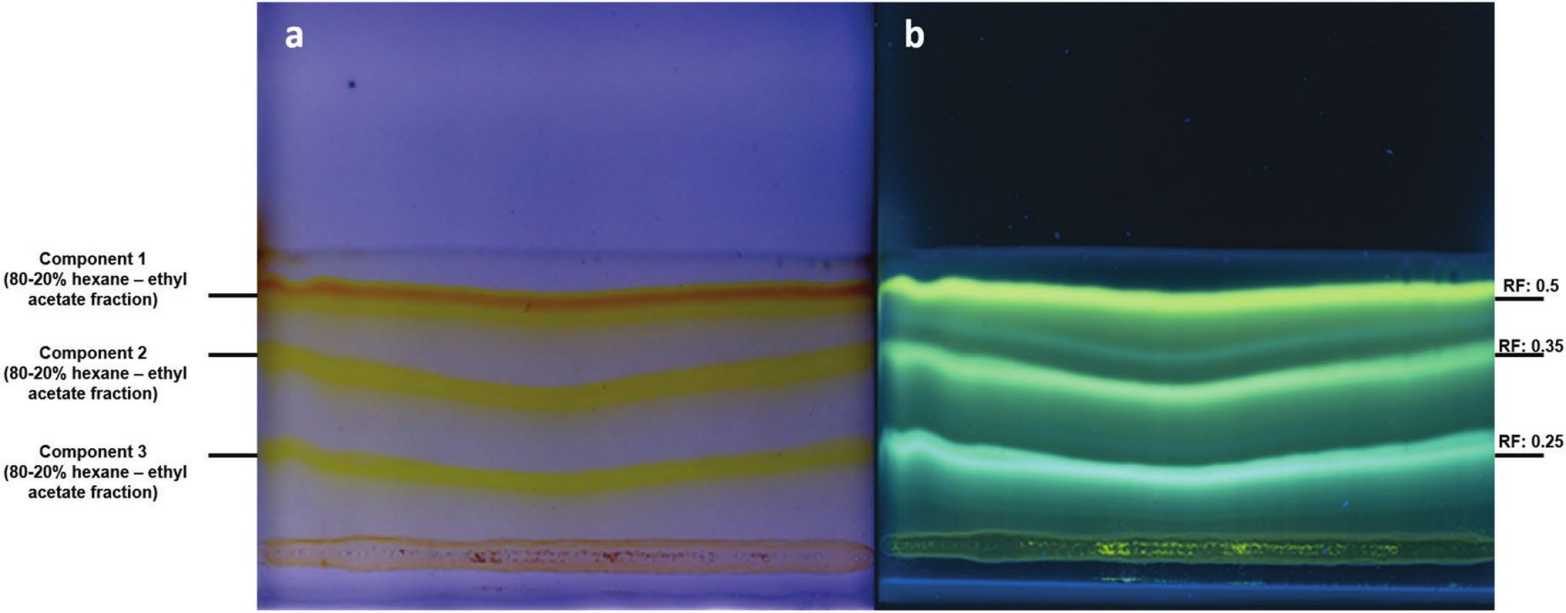
80–20% hexane/ethyl acetate-eluted fractions were sprayed onto a preparative high performance thin layer chromatography plate, and its components were separated using a chloroform/methanol (45:5 v/v) mobile phase. The panel on the left shows the component separation under visible light of possible curcuminoids. The panel on the right shows the same plaque but under UV light at 365 nm. Retention factor for each band corresponds to 0.5, 0.35 and 0.25 respectively

For analysis of the molecules produced on *N. benthamiana* plants transformed with the C4 vector, 15 g of leaf tissue were processed. Each fraction obtained from the eluotropic series was supplemented with a commercial curcuminoid mixture (containing the three curcuminoids) in order to directly visualize and compare the banding pattern of each fraction against the positive control. The results showed that the fraction eluted with 80% hexane–20% ethyl acetate exhibited a banding and separation pattern nearly identical to the same fraction supplemented with the commercial mixture and the positive control (Fig. 5 lanes 6–7 and 24). Based on these findings, this fraction was selected for application onto a preparative TLC plate to isolate its components. Three major bands were observed under visible UV light at 365 nm, with retention factor values of 0.50, 0.35, and 0.25, respectively (Fig. 6a and b). The silica from the separated bands was scraped off the plate and the compounds subsequently extracted and analyzed by mass spectrometry.

### RP-HPLC analysis of compounds extracted from the 80:20 hexane–ethyl acetate fraction

**Fig. 7:**
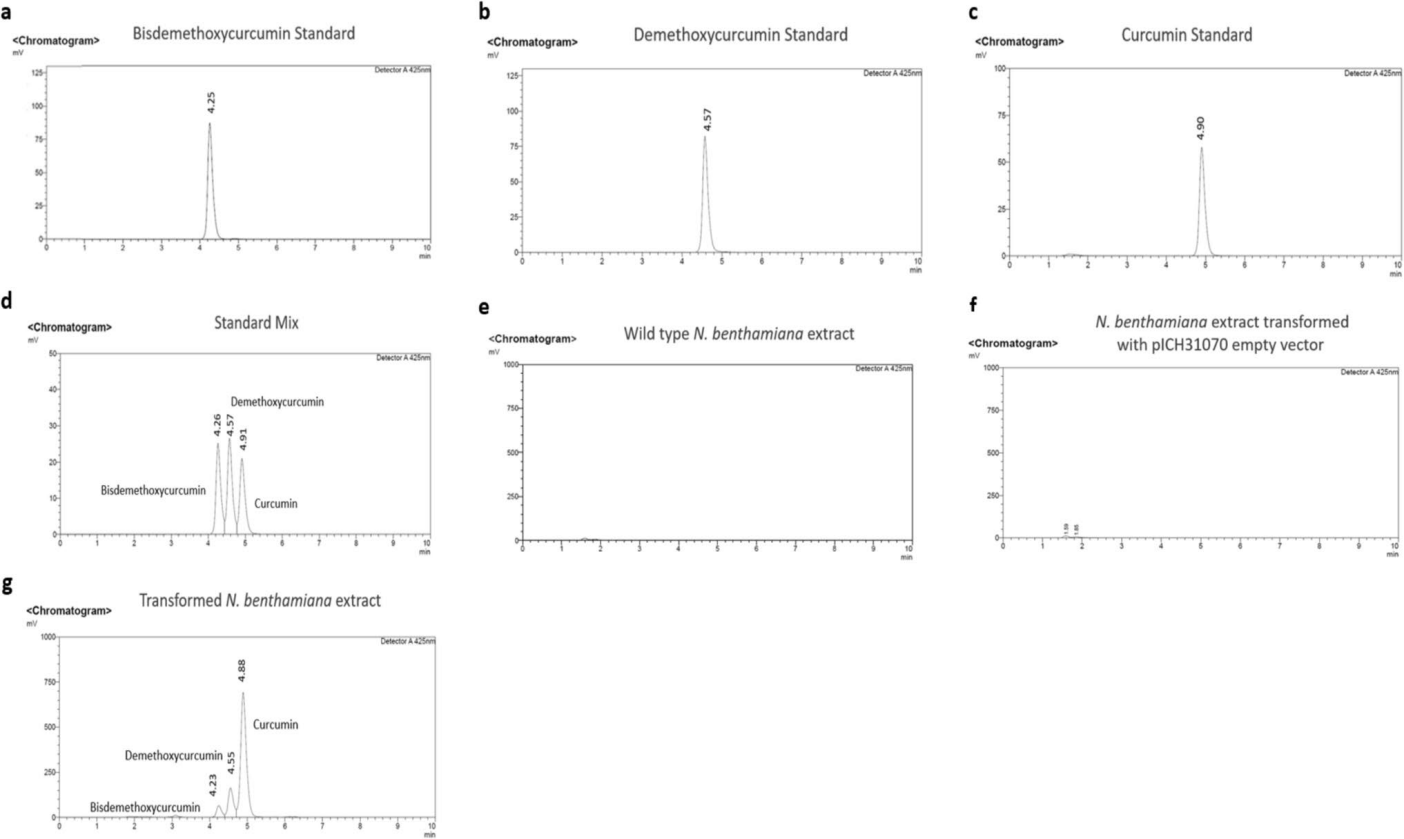
Detection of curcuminoids by RP-HPLC using curcuminoid analytical standards for positive controls (**a**–**c**) and wild type *N. benthamiana* extract for negative control (**e**). A mixture of the three analytical standards was analyzed (**d**) for comparison against C4-transformed plant extract (**g**). Extract of *N. benthamiana* transformed with the empty vector (pICH31070) showed no presence of curcuminoids (**f**)

**Fig. 8:**
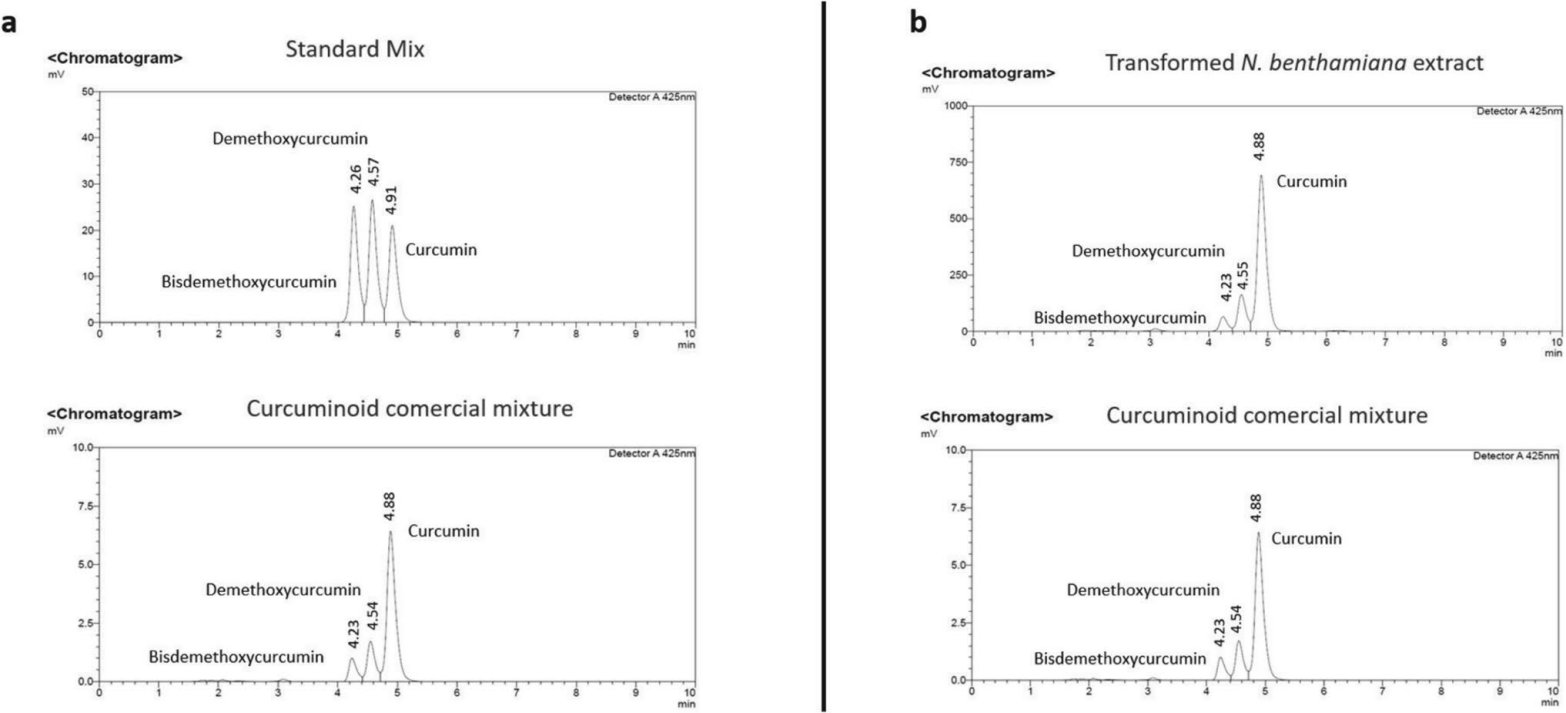
Comparison by RP-HPLC between the retention times of curcuminoids found in the commercial mixture against the mixture of curcuminoid standards (**a**). Comparison between the relative abundance profile between the commercial mixture against *N. benthamiana* transformed extract (**b**)

In order to confirm if the compounds produced in our plants were indeed curcuminoids, commercial standards for curcumin, demethoxycurcumin and bisdemethoxycurcumin were analyzed using RP-HPLC as controls. The retention times of the identified curcuminoids were 4.25 min for bisdemethoxycurcumin, 4.57 min for demethoxycurcumin, and 4.9 min for curcumin (Fig. 7a–c). Given that the extract obtained from the transformed *N. benthamiana* plants was expected to contain all three curcuminoids simultaneously, the three reference standards were combined and analyzed. For this purpose, the standards were mixed at equal concentrations into a single sample in order to evaluate potential changes in retention times; the retention times for all three curcuminoids remained essentially unchanged (Fig. 7d). As negative controls, extracts from wild-type *N. benthamiana* and plants transformed with the empty vector were analyzed under the same conditions; no curcuminoids were detected (Fig. 7e and f). In contrast, the extract from plants transformed with the C4 vector demonstrated the presence of curcumin, demethoxycurcumin and bisdemethoxycurcumin (Fig. 7g). We also wanted to confirm whether the three curcuminoids of interest were present in the commercial mixture that had been used as a positive control. Accordingly, the mixture was analyzed under the same conditions as the mixture of commercial standards to identify the presence of curcumin, demethoxycurcumin, and bisdemethoxycurcumin, as well as to compare their retention times. The results revealed signals corresponding to all three curcuminoids, exhibiting retention times nearly identical to those observed for each standard. The relative abundance of each of the three curcuminoids identified in the commercial mixture was consistent with the values reported in the literature (Fig. 8a). It was also noteworthy that the relative abundance profile of the curcuminoids identified in the commercial mixture was very similar to that observed in the extract from *N. benthamiana* plants transformed with the C4 vector (Fig. 8b). By using RP-HPLC, we were able to confirm the presence of the three main curcuminoids in the transiently transformed plants. Nevertheless, a second analytical approach was employed to obtain a more conclusive confirmation of heterologous curcuminoid production.

### Detection of three different curcuminoids in extracts from *N. benthamiana* plant transiently transformed with the C4 vector by ESI–MS (−)

**Fig. 9:**
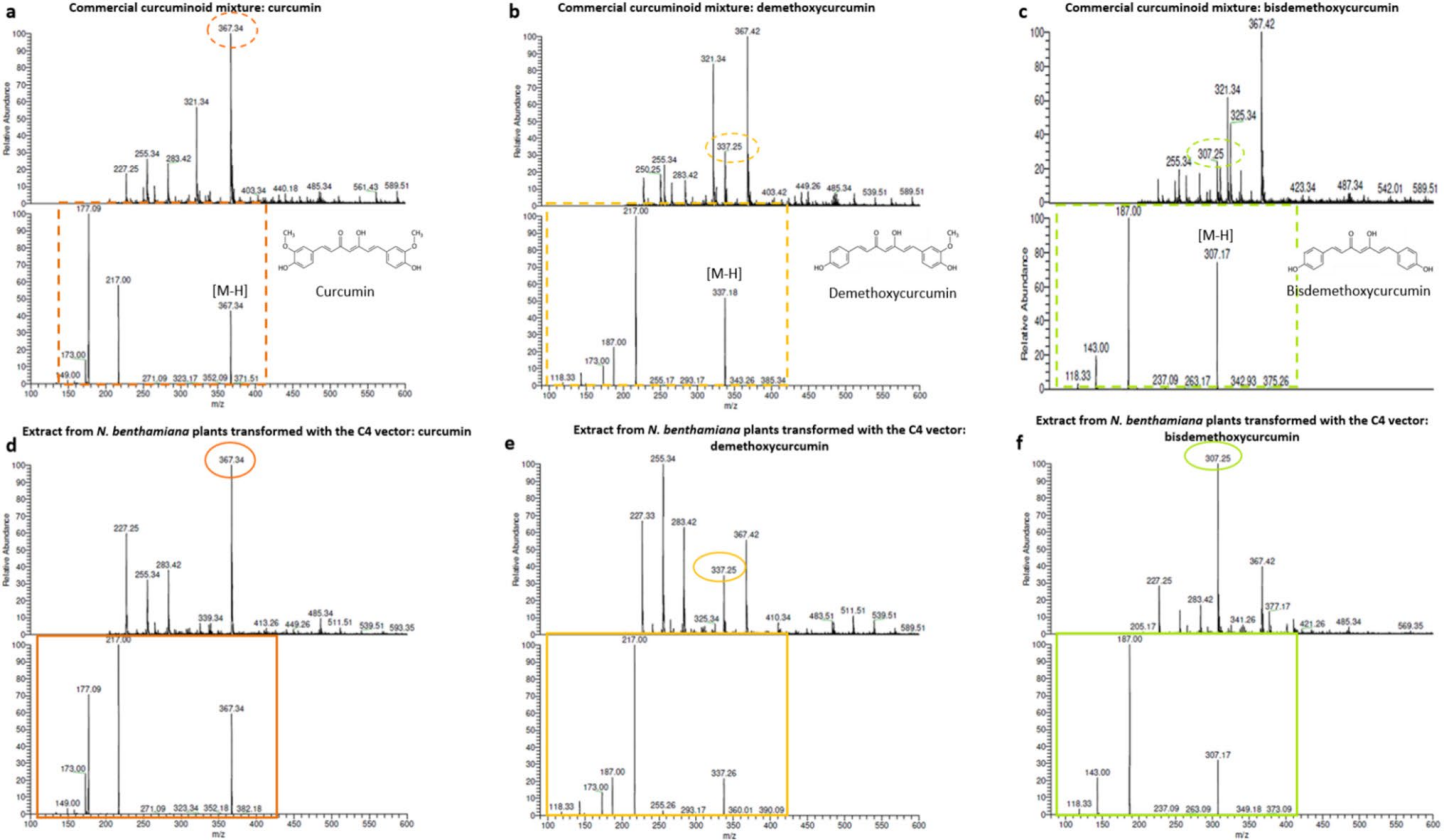
Curcuminoids isolated from commercial mixture by preparative TLC was analyzed by ESI–MS (−). A signal of 367 g/mol, corresponding to deprotonated curcumin (dotted orange circle), was detected. **a** This ion was subjected to collision-induced dissociation to obtain the fragmentation pattern (dotted orange square). **b** and **c** The same protocol was repeated for demethoxycurcumin (dotted yellow circle and dotted yellow square respectively) and bisdemethoxycurcumin (dotted green circle and dotted green square respectively). Curcumin extracted from transformed *N. benthamiana* plants was analyzed by ESI–MS (−) resulting a signal of 367 g/mol, corresponding to deprotonated curcumin (dotted orange circle). **d** This ion was subjected to collision-induced dissociation to obtain a fragmentation spectrum consistent with both the positive control and previously reported data in bibliography. Same protocol was repeated for demethoxycurcumin and bisdemethoxycurcumin produced by transient expression. **e** and **f** Molecular weight and fragmentation spectrum obtained for demethoxycurcumin and bisdemethoxycurcumin were identical in comparison with positive controls

In order to conclusively confirm the presence of curcuminoids in the extract from *N. benthamiana* plants transformed with the C4 vector, ESI–MS (−) analysis was performed. Negative ion mode was selected due to its enhanced efficiency in detecting diarylheptanoids, such as curcuminoids. The three curcuminoids found in the commercial mixture were analyzed individually and its fragmentation spectrum was recorded for comparison with the spectra of curcuminoids produced in *N. benthamiana* transiently transformed plants. Analysis of the commercial mixture revealed the presence of three curcuminoids: (1) curcumin, with a molecular ion corresponding to the deprotonated form of curcumin, at 367 g/mol [M–H^+^]–, fragmentation ions at 217 [C_12_H_11_O_4_–2H^+^], 177 [C_10_H_9_O_3_], 173 [C_11_H_10_O_2_–H^+^], and 149 [C_9_H_9_O_2_]. (2) Demethoxycurcumin, with a molecular ion corresponding to the deprotonated form of demethoxycurcumin at 337 g/mol [M–H^+^]–, fragmentation ions at 217 [C_12_H_11_O_4_–2H^+^], 187 [C_11_H_7_O_3_], 173 [C_11_H_10_O_2_–H^+^], and 118 [C_8_H_6_O]. (3) Bisdemethoxycurcumin, with a molecular ion corresponding to its deprotonated form at 307 g/mol [M–H^+^]–, 187 [C_11_H_7_O_3_], 143 [C_10_H_8_O–H^+^], and 118 [C_8_H_6_O]. The stable chemical structures of the three diarylheptanoids exhibited a mass difference of 30 g/mol between curcumin–demethoxycurcumin and demethoxycurcumin–bisdemethoxycurcumin, suggesting the loss of a methoxy group. These obtained values and fragmentation patterns matched with previously reported spectra in the literature for the three curcuminoids (Jiang et al. 2006; Herebian et al. 2009; Lee et al. 2014) (Fig. 9a–c). The fragmentation pattern of the compounds obtained from C4 transiently transformed plants were compared against each curcuminoid from the commercial mixture showing identical fragmentation patterns (Fig. 9d–f) These results provide solid evidence for the successful heterologous production of curcumin in *N. benthamiana* by transient expression.

### Quantification of curcuminoids produced in *N. benthamiana* plants transformed with the C4 vector

**Fig. 10:**
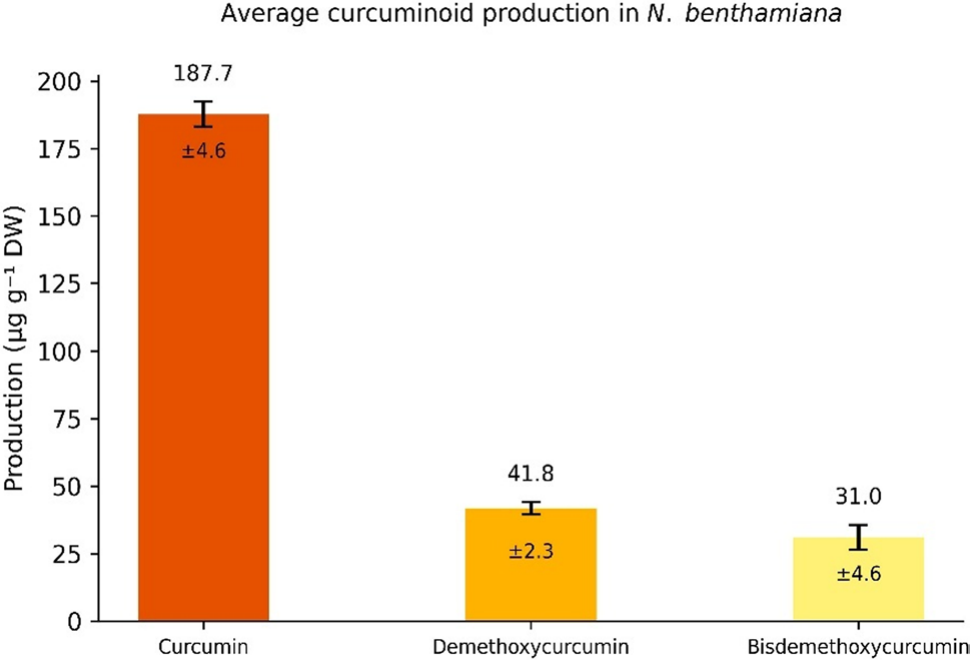
Yields obtained for each curcuminoid in *N. benthamiana* transiently transformed plants with the C4 vector. The results were obtained after three different performed transient transformation experiments. Each bar represents the mean value obtained from three independent transient transformation experiments. Error bars indicate standard deviation (± SD; *n* = 10 plants for each transformation experiment)

Curcuminoids produced in *N. benthamiana* by transient expression with the C4 vector were quantified by RP-HPLC as described in Materials and methods. Figure 10 shows the mean production of each curcuminoid obtained in three different transient transformation experiments. The yield obtained was 187.7 ± 4.6 µg/g DW for curcumin, 41.8 ± 2.3 µg/g DW for demethoxycurcumin and 31 ± 4.6 µg/g DW for bisdemethoxycurcumin.

## Discussion

In this work we established by two different methodologies that curcuminoids can be produced in *N. benthamiana* plants by simultaneous expression of DCS, CURS1, CURS2 and CURS3 providing definitive structural evidence for the three curcuminoids production (Fig. 9 and Suppl. Table S1). Taken together, these results constitute strong chemical validation that the introduced polyketide synthases machinery is functional within the *N. benthamiana* metabolome (Katsuyama et al. 2009a, b).

Beyond verification of product identity, the metabolite proportions observed in the heterologous plant system, are very informative. The predominance of curcumin relative to demethoxy- and bisdemethoxycurcumin in our engineered *N. benthamiana*, mirrors the natural turmeric composition (77:18:5), suggesting that the catalytic preferences and substrate selectivity of CURS isoforms (CURS1–3) are preserved when expressed in the heterologous plant host and that endogenous precursor fluxes are sufficient to support the native distribution (Figs. 7 and 8). This is consistent with prior characterizations of CURS enzyme specificities in the commercial mixture (Katsuyama et al. 2009a, b). In contrast, there are no reports in any curcuminoid producing platform in which the proportion of curcuminoids mimics the natural system found in *C. longa*.

Comparative evaluation with microbial production systems highlights the advantages and the current limitations of production in plants. Microbial platforms can achieve high titers (up to 696 mg·L^−1^ of curcumin in a pilot fermentation) via intensive pathway optimization, gene knockouts, efflux strategies, and operations engineering (Chen et al. 2024). Similarly, *Y. lipolytica* has been engineered with several genes from the phenylpropanoid route, including the curcuminoid synthase gene from *O. sativa* but only bisdemethoxycurcumin was detectable in the engineered lines (Palmer et al. 2020). Nevertheless, such successes have required extensive re-organization of central metabolism, repeated rounds of optimization, and targeted interventions to increase malonyl-CoA and to mitigate product/intermediate toxicity—challenges that are both technically demanding and restrictive for host fitness (Liao et al. 2016).

A critical constraint in microbial production of plant polyketides is intracellular malonyl-CoA availability. Malonyl-CoA is a highly regulated metabolite predominantly committed to fatty acid biosynthesis; increasing its pool availability, typically necessitates overexpression or deregulation of acetyl-CoA carboxylase (ACC), elimination of competing routes, or introduction of novel malonyl-CoA generating pathways—each intervention carrying tradeoffs for growth and metabolic robustness (Liao et al. 2016). In addition, intermediates such as ferulic acid can impose cytotoxic stress on bacterial and yeast hosts unless detoxification or export strategies are implemented (Parke and Ornston 2004; Rodrigues et al. 2020; Combes et al. 2022; Rodríguez-Ochoa et al. 2022). Thus, achieving both high titer and economic process stability in microbes remains a resource-intensive engineering problem. In this context, the heterologous plant system production approach reported here offers distinct practical and biological advantages. Plants inherently possess the phenylpropanoid pathway and associated enzymatic repertoire that generate the CoA starters required for CURS activity, thereby reducing the number of heterologous genes necessary to reconstitute curcuminoid biosynthesis (in our case, only the four turmeric enzymes were required) and avoiding extensive rewiring of central carbon metabolism.

Regulatory and biosafety considerations also differ between plant and microbial production; for example, plant-based transient expression often entails lower containment complexity and reduced cross-pathogen concerns compared with large-scale cultivation of genetically modified microbes. These properties collectively make the plant chassis an attractive complementary route for producing complex, multi-step natural products that rely on endogenous pools of phenylpropanoid precursors (Van Beirs et al. 2025). Nevertheless, we are aware that yields obtained in this study remain lower than industrial microbial titers reported in the literature and may require further optimization to become commercially viable. Although the yield obtained in our system is lower than that naturally occurring in turmeric rhizomes (3–4% DW), it is important to emphasize the markedly reduced production time and the controlled conditions in which the plants are grown. Conventional cultivation of turmeric requires approximately 12–18 months to reach harvest, without considering potential yield losses associated with climatic conditions or pathogen contamination. Producers of curcuminoids are very careful that the rhizomes that they utilize must be in excellent health (Rodrigues et al. 2015). In contrast, our approach enables the production of an acceptable yield within approximately 9–10 weeks, highlighting the potential of this system as a rapid and flexible alternative for curcuminoid biosynthesis and keeping the plants in controlled conditions, free of pathogens.

On the other hand, it is important to emphasize the need to develop further strategies aimed at improving production systems for molecules of interest. Martí-Botella (2022) attempted production of curcumin in *N. benthamiana*, achieving a yield of approximately 22 ± 4 µg/g DW. In our case, by employing a deconstructed viral system and two additional enzymes (CURS1 and CURS2), we were able to produce 187.7 ± 4.6 µg/g DW of curcumin, 41.8 ± 2.3 µg/g DW of demethoxycurcumin and 31 ± 4.6 µg/g DW of bisdemethoxycurcumin. Notably, our curcumin yield is very similar to that reported by Singh et al. (2021) following stable transformation of *A. belladonna* hairy roots, which resulted in 180.62 ± 4.7 µg/g DW of curcumin. Together, these findings highlight the importance of exploring diverse approaches, as each strategy can lead to different production levels while offering distinct advantages.

When comparing plants as heterologous platforms for curcuminoid expression, the most immediate precedent is represented by the production of curcumin in *A. belladonna* (Singh et al. 2021). Although both systems achieved a very similar level of curcumin production, our system is not subject to the regulatory frameworks over transgenic organisms. Potential strategies to increase the yield in plants include: metabolic channeling through the desired enzymes, modulation of endogenous precursor supply or long-term metabolite accumulation. These engineering concepts are supported by prior examples in both plant and microbial systems, where precursor pathway enhancement or compartmentalization substantially improved malonyl-CoA-derived product titers (Liao et al. 2016).

Finally, the biosynthetic fidelity exhibited by CURS enzymes in *N. benthamiana* (product ratio similar to *C. longa*) is encouraging for pathway transferability and suggests that future efforts might focus on process and host optimization rather than reengineering CURS substrate selectivity. Our data provide evidence that the expressed enzymes were present at sufficient levels and in apparent, appropriate stoichiometry to sustain pathway functionality. In conclusion, the data presented here validate *N. benthamiana* as a viable heterologous chassis for curcuminoid production and provide a guideline for engineering strategies on future studies.

## Supplementary Information

Below is the link to the electronic supplementary material.

Supplementary file1 (DOCX 134 KB)

## Data Availability

All data generated or analyzed in this study are presented in this published article.

## Abbreviations:

CURS1–3: Curcumin synthases 1–3
CUS: Curcuminoid synthase
DCS: Diketide–CoA synthase
DW: Dry weight
ESI–MS ( −): Electrospray ionization–mass spectrometry (negative ion mode)
RP-HPLC: Reversed-phase high-performance liquid chromatography
