# Supplementary material for: Metabolic engineering of Nicotiana benthamiana for production of curcuminoids
Source: Planta. 2026 May 11;263(6):152. doi: 10.1007/s00425-026-05014-x (PMC13161023; doi:10.1007/s00425-026-05014-x)
Supplement: Supplementary file 1 — Supplementary file1 (DOCX 134 KB) [file 425_2026_5014_MOESM1_ESM.docx]

**Metabolic Engineering of Nicotiana benthamiana for Production of Curcuminoids**

Rafael González-Castro, Enrique Ramírez Chávez, Jorge Molina Torres, Brisia Alejandra Aguilar-Barragán, Miguel A Gómez Lim*

Centro de Investigación y Estudios Avanzados del Instituto Politécnico Nacional, Unidad Irapuato. Irapuato, Guanajuato, México, CP 36821

* Corresponding author: Miguel A. Gómez Lim ([miguel.gomez@cinvestav.mx](mailto:miguel.gomez@cinvestav.mx))

**Supplementary materials**

**C4 vector nucleotide sequence**

GCTTGAAGACTAGGCGTGGTGCGCACGATAGCGCATAGTGTTTTTCTCTCCACTTGAATCGAAGAGATAGACTTACGGTGTAAATCCGTAGGGGTGGCGTAAACCAAATTACGCAATGTTTTGGGTTCCATTTAAATCGAAACCCCTTATTTCCTGGATCACCTGTTAACGCACGTTTGACGTGTATTACAGTGGGAATAAGTAAAAGTGAGAGGTTCGAATCCTCCCTAACCCCGGGTAGGGGCCCAGCGGCCGCTCTAGCTAGAGTCAAGCAGATCGTTCAAACATTTGGCAATAAAGTTTCTTAAGATTGAATCCTGTTGCCGGTCTTGCGATGATTATCATATAATTTCTGTTGAATTACGTTAAGCATGTAATAATTAACATGTAATGCATGACGTTATTTATGAGATGGGTTTTTATGATTAGAGTCCCGCAATTATACATTTAATACGCGATAGAAAACAAAATATAGCGCGCAAACTAGGATAAATTATCGCGCGCGGTGTCATCTATGTTACTAGATCGACTAGTGAGCTCTGCATCCACCCCAGTACATTAAAAACGTCCGCAATGTGTTATTAAGTTGTCTAAGCGTCAATTTGTTTACACCACAATATATCCTGCCACCAGCCAGCCAACAGCTCCCCGACCGGCAGCTCGGCACAAAATCACCACTCGATACAGGCAGCCCATCAGTCAGATCAGGATCTCCTTTGCGACGCTCACCGGGCTGGTTGCCCTCGCCGCTGGGCTGGCGGCCGTCTATGGCCCTGCAAACGCGCCAGAAACGCCGTCGAAGCCGTGTGCGAGACACCGCGGCCGCCGGCGTTGTGGATACCTCGCGGAAAACTTGGCCCTCACTGACAGATGAGGGGCGGACGTTGACACTTGAGGGGCCGACTCACCCGGCGCGGCGTTGACAGATGAGGGGCAGGCTCGATTTCGGCCGGCGACGTGGAGCTGGCCAGCCTCGCAAATCGGCGAAAACGCCTGATTTTACGCGAGTTTCCCACAGATGATGTGGACAAGCCTGGGGATAAGTGCCCTGCGGTATTGACACTTGAGGGGCGCGACTACTGACAGATGAGGGGCGCGATCCTTGACACTTGAGGGGCAGAGTGCTGACAGATGAGGGGCGCACCTATTGACATTTGAGGGGCTGTCCACAGGCAGAAAATCCAGCATTTGCAAGGGTTTCCGCCCGTTTTTCGGCCACCGCTAACCTGTCTTTTAACCTGCTTTTAAACCAATATTTATAAACCTTGTTTTTAACCAGGGCTGCGCCCTGTGCGCGTGACCGCGCACGCCGAAGGGGGGTGCCCCCCCTTCTCGAACCCTCCCGGCCCGCTAACGCGGGCCTCCCATCCCCCCAGGGGCTGCGCCCCTCGGCCGCGAACGGCCTCACCCCAAAAATGGCAGCGCTGGCCAATTCGTGCGCGGAACCCCTATTTGTTTATTTTTCTAAATACATTCAAATATGTATCCGCTCATGAGACAATAACCCTGATAAATGCTTCAATAATATTGAAAAAGGAAGAGTATGGCTAAAATGAGAATATCACCGGAATTGAAAAAACTGATCGAAAAATACCGCTGCGTAAAAGATACGGAAGGAATGTCTCCTGCTAAGGTATATAAGCTGGTGGGAGAAAATGAAAACCTATATTTAAAAATGACGGACAGCCGGTATAAAGGGACCACCTATGATGTGGAACGGGAAAAGGACATGATGCTATGGCTGGAAGGAAAGCTGCCTGTTCCAAAGGTCCTGCACTTTGAACGGCATGATGGCTGGAGCAATCTGCTCATGAGTGAGGCCGATGGCGTCCTTTGCTCGGAAGAGTATGAAGATGAACAAAGCCCTGAAAAGATTATCGAGCTGTATGCGGAGTGCATCAGGCTCTTTCACTCCATCGACATATCGGATTGTCCCTATACGAATAGCTTAGACAGCCGCTTAGCCGAATTGGATTACTTACTGAATAACGATCTGGCCGATGTGGATTGCGAAAACTGGGAAGAAGACACTCCATTTAAAGATCCGCGCGAGCTGTATGATTTTTTAAAGACGGAAAAGCCCGAAGAGGAACTTGTCTTTTCCCACGGCGACCTGGGAGACAGCAACATCTTTGTGAAAGATGGCAAAGTAAGTGGCTTTATTGATCTTGGGAGAAGCGGCAGGGCGGACAAGTGGTATGACATTGCCTTCTGCGTCCGGTCGATCAGGGAGGATATCGGGGAAGAACAGTATGTCGAGCTATTTTTTGACTTACTGGGGATCAAGCCTGATTGGGAGAAAATAAAATATTATATTTTACTGGATGAATTGTTTTAGCTGTCAGACCAAGTTTACTCATATATACTTTAGATTGATTTAAAACTTCATTTTTAATTTAAAAGGATCTAGGTGAAGATCCTTTTTGATAATCTCATGACCAAAATCCCTTAACGTGAGTTTTCGTTCCACTGAGCGTCAGACCCCGTAGAAAAGATCAAAGGATCTTCTTGAGATCCTTTTTTTCTGCGCGTAATCTGCTGCTTGCAAACAAAAAAACCACCGCTACCAGCGGTGGTTTGTTTGCCGGATCAAGAGCTACCAACTCTTTTTCCGAAGGTAACTGGCTTCAGCAGAGCGCAGATACCAAATACTGTCCTTCTAGTGTAGCCGTAGTTAGGCCACCACTTCAAGAACTCTGTAGCACCGCCTACATACCTCGCTCTGCTAATCCTGTTACCAGTGGCTGCTGCCAGTGGCGATAAGTCGTGTCTTACCGGGTTGGACTCAAGACGATAGTTACCGGATAAGGCGCAGCGGTCGGGCTGAACGGGGGGTTCGTGCACACAGCCCAGCTTGGAGCGAACGACCTACACCGAACTGAGATACCTACAGCGTGAGCTATGAGAAAGCGCCACGCTTCCCGAAGGGAGAAAGGCGGACAGGTATCCGGTAAGCGGCAGGGTCGGAACAGGAGAGCGCACGAGGGAGCTTCCAGGGGGAAACGCCTGGTATCTTTATAGTCCTGTCGGGTTTCGCCACCTCTGACTTGAGCGTCGATTTTTGTGATGCTCGTCAGGGGGGCGGAGCCTATGGAAAAACGCCAGCAACGCGGCCTTTTTACGGTTCCTGGCAGATCCTAGATGTGGCGCAACGATGCCGGCGACAAGCAGGAGCGCACCGACTTCTTCCGCATCAAGTGTTTTGGCTCTCAGGCCGAGGCCCACGGCAAGTATTTGGGCAAGGGGTCGCTGGTATTCGTGCAGGGCAAGATTCGGAATACCAAGTACGAGAAGGACGGCCAGACGGTCTACGGGACCGACTTCATTGCCGATAAGGTGGATTATCTGGACACCAAGGCACCAGGCGGGTCAAATCAGGAATAAGGGCACATTGCCCCGGCGTGAGTCGGGGCAATCCCGCAAGGAGGGTGAATGAATCGGACGTTTGACCGGAAGGCATACAGGCAAGAACTGATCGACGCGGGGTTTTCCGCCGAGGATGCCGAAACCATCGCAAGCCGCACCGTCATGCGTGCGCCCCGCGAAACCTTCCAGTCCGTCGGCTCGATGGTCCAGCAAGCTACGGCCAAGATCGAGCGCGACAGCGTGCAACTGGCTCCCCCTGCCCTGCCCGCGCCATCGGCCGCCGTGGAGCGTTCGCGTCGTCTCGAACAGGAGGCGGCAGGTTTGGCGAAGTCGATGACCATCGACACGCGAGGAACTATGACGACCAAGAAGCGAAAAACCGCCGGCGAGGACCTGGCAAAACAGGTCAGCGAGGCCAAGCAGGCCGCGTTGCTGAAACACACGAAGCAGCAGATCAAGGAAATGCAGCTTTCCTTGTTCGATATTGCGCCGTGGCCGGACACGATGCGAGCGATGCCAAACGACACGGCCCGCTCTGCCCTGTTCACCACGCGCAACAAGAAAATCCCGCGCGAGGCGCTGCAAAACAAGGTCATTTTCCACGTCAACAAGGACGTGAAGATCACCTACACCGGCGTCGAGCTGCGGGCCGACGATGACGAACTGGTGTGGCAGCAGGTGTTGGAGTACGCGAAGCGCACCCCTATCGGCGAGCCGATCACCTTCACGTTCTACGAGCTTTGCCAGGACCTGGGCTGGTCGATCAATGGCCGGTATTACACGAAGGCCGAGGAATGCCTGTCGCGCCTACAGGCGACGGCGATGGGCTTCACGTCCGACCGCGTTGGGCACCTGGAATCGGTGTCGCTGCTGCACCGCTTCCGCGTCCTGGACCGTGGCAAGAAAACGTCCCGTTGCCAGGTCCTGATCGACGAGGAAATCGTCGTGCTGTTTGCTGGCGACCACTACACGAAATTCATATGGGAGAAGTACCGCAAGCTGTCGCCGACGGCCCGACGGATGTTCGACTATTTCAGCTCGCACCGGGAGCCGTACCCGCTCAAGCTGGAAACCTTCCGCCTCATGTGCGGATCGGATTCCACCCGCGTGAAGAAGTGGCGCGAGCAGGTCGGCGAAGCCTGCGAAGAGTTGCGAGGCAGCGGCCTGGTGGAACACGCCTGGGTCAATGATGACCTGGTGCATTGCAAACGCTAGGGCCTTGTGGGGTCAGTTCCGGCTGGGGGTTCAGCAGCCAGCGCCTGATCTGGGGAACCCTGTGGTTGGCACATACAAATGGACGAACGGATAAACCTTTTCACGCCCTTTTAAATATCCGATTATTCTAATAAACGCTCTTTTCTCTTAGGTTTACCCGCCAATATATCCTGTCAAACACTGATAGTTTAAACTGAAGGCGGGAAACGACAATCTGATCTAAGCTAGGGTACCTCGAAGCCGCGGTGCGGGTGCCAGGGCGTGCCCTTGGGCTCCCCGGGCGCGTACTCCACCTCACCCATCTTTTATTACATGTTTGAACTTCAACAATTTATGACTTTTTGTTCTTATTGTTGCaggtatggccaacctccacgcgttgcgcagggagcagagggctcaaggtcctgccaccatcatggccatcgggaccgccacccctcccaacctctacgagcagagcaccttcccggacttctacttccgcgtcaccaactccgacgacaagcaggagctcaagaaaaagttccgccgcatgtgcgagaagacgatggtgaagaagcggtacctgcacttgaccgaggagatcctgaaggagaggcccaagctctgctcctacaaggaggcgtcgttcgacgaccggcaggacatcgtggtggaggagataccgagattggctaaggaagcggcggagaaggccatcaaggagtgggggcggcccaaatcggagatcacccacctggtcttctgctccatcagcgggatcgacatgcccggcgccgactaccgcctcgccacgctcctcgggctccctctcaccgtcaaccgcctcatgatctacagccaggcctgccacatgggcgccgccatgctccgcatcgccaaggacctcgccgagaacaacaggggcgcgcgcgtgctggtggtcgcctgcgagatcaccgtgctcagcttccgcggcccgaacgagSrvcntCstanttySbttaGnsynthssGnsynthssGnsynthssrdrSbttaShngHandngtTtaggcgacttcgaggcgctcgcggggcaggccggcttcggcgacggcgcgggggccgtcgtcgtcggggccgacccgctggaaggaattgaaaaacccatctacgagatcgcggcggcgatgcaggagacggtggcggagagccagggggcggtgggcggccacctgcgggccttcggctggacgttctacttcctgaaccagctgccggcgatcatcgccgacaacctcgggaggagcctggagcgggcgttggcgccgctgggggtgagggagtggaacgacgtcttctgggtggcgcacccgggcaactgggccatcattgacgccatcgaagccaagctgcagctgagcccggacaagctcagcaccgcccgccacgtcttcacagagtacggcaacatgcagagcgccaccgtgtacttcgtgatggatgagctgaggaagcggtcggcggtggaggggcggagcaccaccggcgacggcttgcagtggggagttctcctcggttttgggccgggcctcagcatcgaaaccgttgtactgcgcagtatgccactgATGTTGCAAACTGAAGAAGATAAAAAACCTTCCGGAGATCAGGCAGCTCACATAAATTTAAAGGTTAAAGGCCAAGATGGAAACGAGGTATTTTTTCGAATTAAACGATCTACTCAATTAAAGAAATTAATGAATGCATATTGTGATAGGCAATCAGTTGACTTTAACTCAATAGCTTTTCTGTTTGACGGGAGGAGGCTTCGTGCCGAGCAAACTCCTGATGAACTAGAGATGGAAGACGGTGATGAGATTGATGCTATGCTCCACCAGACTGGCGGAatggcgatgatcagcttgcaggcgatgcgcaaggcgcagagagctcaaggtccggccaccatcttggccgtcggcaccgccaacccgcccaatctctacgagcaggacacgtatcccgactactacttccgcgtcaccaactccgagcacaagcaggagctcaagaacaagttccgcctcatgtgcgagaagacgatggtgaagaggcggtatctttacctgacgccggagatcctgaaggagcggccgaagctgtgctcgtacatggagccgtcgttcgacgaccggcaggacatcgtggtggaggaggtgccgaagctggccgcggaggcagcggagaacgccatcaaggagtggggcggcgacaagtccgccatcacccacctggtcttctgctccatcagcggcatcgacatgcccggagctgactaccgcctcgcccagctcctcggactcccgctcgccgtcaaccgcctgatgctctacagccaggcctgccacatgggcgccgccatgctgcgcatagccaaggacatcgccgagaacaaccgctccgcgcgcgtcctcgtcgtcgcctgcgagatcaccgtgctcagcttccgcggcccggacgagcgcgacttccaggcgctggccggccaggccggcttcggggacggcgccggcgcgatgatcgtcggggccgaccccgtcctcggcgtcgagcggccgctctaccacatcatgtcggcgactcagacgacggtaccggagagcgagaaggcggtggggggccacctccgcgaggtggggctgaccttccacttcttcaaccagctgccggcgatcatcgccgacaacgtggggaacagcctggcggaggcgttcgaaccgatcgggatcaaggactggaacaacatcttctgggtggcgcacccgggcaactgggccatcatggacgccatcgaaaccaagctgggcctggaacagagcaagctggccaccgcacgccacgtcttctccgagttcggcaacatgcagagcgccaccgtctacttcgtgatggacgagctcaggaaacggtcggcggcggagaaccgggcgaccaccggcgacgggctccggtggggcgtgctcttcggcttcggcccgggcatcagcatcgaaaccgtcgtgctccaaagcgtgccgcttATGCAGATCTTTGTTAAAACCCTCACTGGTAAAACAATTACACTGGAAGTCGAATCATCAGATACAATTGATAACGTAAAAGCTAAAATACAAGATAAAGAAGGAATCCCTCCAGATCAACAAAGGCTGATATTTGCAGGTAAGCAACTGGAAGATGGACGAACCCTTGCCGATTATAACATTCAAAAAGAATCTACTCTTCATCTTGTTTTAAGACTACGAGGAGGTatgggcagcctgcaggcgatgcgcagggcacagcgggctcaaggcccggccaccatcatggctgtcggcacctccaacccgcctaacctctacgagcagacctcctaccccgacttctatttccgcgtcaccaactccgaccacaagcacgcgctcaagaacaaattccgtgttatctgtgagaagacgaaggtgaaaagacggtacttgcacttgacggaggagatcctgaagcagaggcccaagctctgctcctacatggagccctccttcgacgatcggcaggacatcgtggtggaggagataccaaagctggcgaaggaggcggcggagaaggcgatcaaggagtggggccgccccaagtcggagatcacccacttggtgttctgctccatcagcggtatcgacatgcccggcgccgattaccgcctcgccaccctcctcggcctccccctgtccgtcaaccgcctcatgctctacagccaggcctgccacatgggcgcgcagatgctgcgcatagccaaggacctcgcggagaacaaccggggcgcgcgcgtcctggccgtctcctgcgaaatcaccgtgctcagcttccgcggtccggacgcgggcgacttcgaggccctcgcgtgtcaggccggcttcggcgacggtgccgctgccgtcgtcgtcggggccgaccccctcccgggcgtcgagaggcccatctacgagatcgcggcggcgatgcaggaaacggtgccggagagcgagagggcggtggggggccacctgagggaaatcggctggaccttccacttcttcaaccagcttccgaagctgatcgcggaaaacatcgagggcagcctggcgcgggcgttcaagccgctggggatcagcgagtggaacgacgtgttctgggtggcgcacccggggaactggggcatcatggacgccatcgaaaccaagctggggctggaacaggggaagctcgccacggcgcgccacgtcttcagcgagtacggaaacatgcagagcgccaccgtgtacttcgtgatggacgaggtgaggaagcggtcggcggcggaggggcgggccaccaccggcgaaggcctggagtggggagtgctgtttgggtttggcccaggcctcaccatagaaactgtcgtgctacgcagtgtaccattaccgATGTTGCAGACCGAAGAAGATAAGAAACCCTCAGGTGACCAAGCTGCACATATCAACTTAAAAGTTAAAGGCCAGGATGGCAATGAAGTTTTCTTCAGGATCAAGAGGTCTACACAATTAAAGAAACTAATGAACGCATATTGTGACCGTCAATCTGTTGATTTTAATTCAATTGCATTTCTGTTTGATGGTCGAAGACTCAGGGCAGAACAGACTCCCGATGAATTGGAAATGGAAGACGGAGATGAAATTGATGCTATGTTACATCAAACTGGGGGAatggaagcgaacggctaccgcataactcacagcgccgacgggccggcgacgatcttggccatcggcaccgccaaccccaccaacgtcgtcgatcagaacgcttatcccgacttctatttccgggtcaccaactccgagtatctgcaggaactcaaagccaagtttaggcgcatctgtgagaaagcggccatcaggaagaggcacttgtacttgactgaggagattttgcgggagaatcctagcttgctggctcccatggcgccgtcgttcgacgcgcggcaggcgatcgtggtggaggcggtgccgaagctggcgaaggaggcggcggagaaggcgatcaaggagtggggccgccccaaatcggacatcacgcacctcgtcttctgctccgcgagcggaatcgacatgcccggctccgacctgcagcttctcaagctgctcgggctcccgccgagcgtcaatcgcgtcatgctctacaacgtcgggtgccacgccggtggcaccgccctccgcgtcgccaaggacctcgcggagaacaaccgcggcgcgcgggtgctcgccgtctgctccgaggtcaccgtgctctcctaccgcggcccccaccccgcccacatcgagagcctcttcgtccaagctctgtttggcgacggcgccgccgcgctcgtggtcgggtccgaccccgtcgatggcgtcgagcgccccatcttcgaaatcgcctcggcatcccaagtgatgcttccggagagcgcagaggcggtgggcggccacctccgcgaaattgggctgaccttccacctcaagagccagcttccgtcgatcatcgcgagcaacatcgagcagagcctgacgactgcgtgctcgccgctggggctgtcggactggaaccagctgttctgggcggttcaccccggcggccgagcgatcctggaccaggtggaggcgcggctcggactggagaaggaccggctcgccgcgacgcggcacgtactcagcgagtacggcaacatgcagagcgccacggtgctgttcatcctggacgagatgcggaaccgctcggctgcggagggccacgccaccaccggcgaggggctcgactggggcgtgctgttgggcttcggcccgggactctccatcgaaaccgtcgtcctccatagttgcagactgaactagtaa

**Identification of potential molecules based on the fragmentation patterns obtained by ESI-MS (-)**

Based on the fragmentation patterns obtained upon applying energy to the curcuminoids produced in N. benthamiana plants, we have suggested different curcuminoid-derived molecules that could explain the observed molecular weights (**Table S1**). Not all of the obtained fragments can be seen in the three curcuminoids, nevertheless, the vast majority of the fragmentation spectrum can be accounted for by the structures.

**
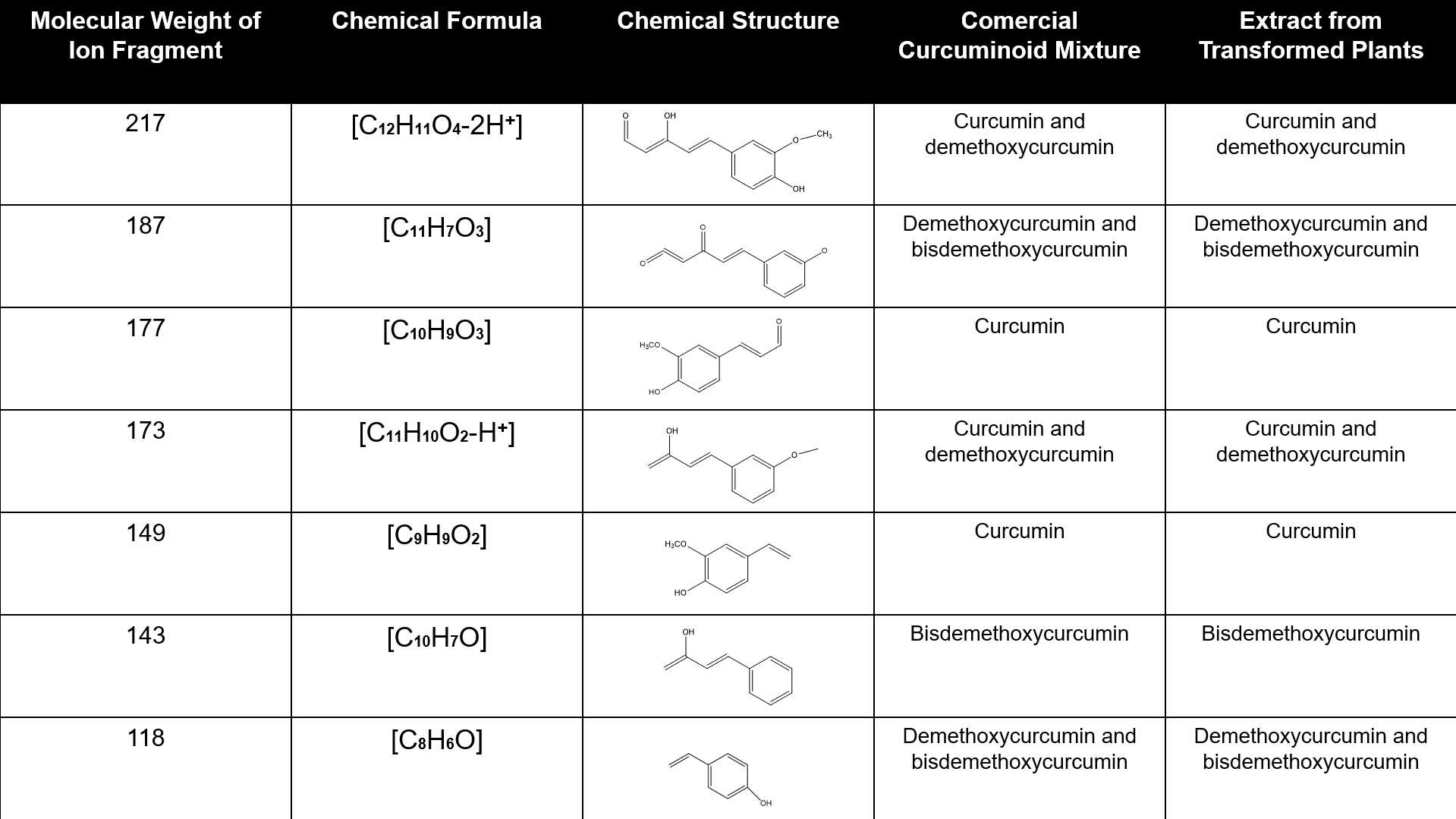
**

Table S1 Possible curcuminoid derived molecules detected in ESI-MS (-) fragmentation patterns. Image was created using ACD/ChemSketch Program for molecular structures
